# Supplementary material for: Integrative Analysis of N6-Methyladenosine-Related Enhancer RNAs Identifies Distinct Prognosis and Tumor Immune Micro-Environment Patterns in Head and Neck Squamous Cell Carcinoma
Source: Cancers (Basel). 2022 Sep 25;14(19):4657. doi: 10.3390/cancers14194657 (PMC9563840; doi:10.3390/cancers14194657)
Supplement: Supplementary file 1 [file cancers-14-04657-s001.zip › Figure S1 and Tables S1 and S2.pdf]

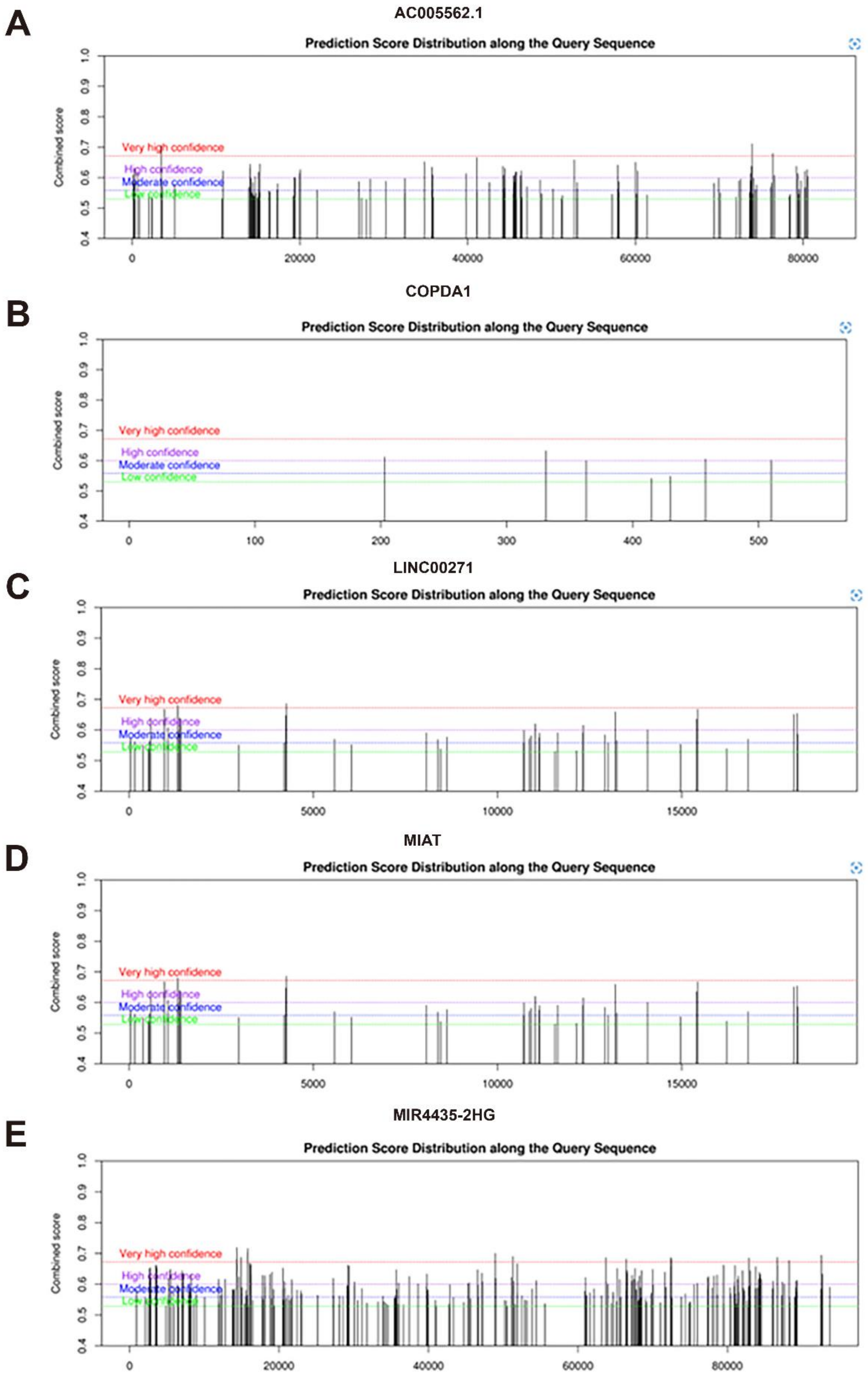

Figure S1. Using the SRAMP website to predict the m6A sites and scores on the RNA sequences of AC005562.1 **(A)**, COPDA1 **(B)**, LINC00271 **(C)**, MIAT **(D)**, MIR4435-2HG **(E)**, respectively.

Table S1. SiRNA sequences.

Gene (Human)	Sense (5′-3′)	Antisense (5′-3′)
siFTO-1	ACACUUGGCUCCCUUAUCUTT	AGAUAAGGGAGCCAAGUGUTT
siFTO-2	GUGGCAGUGACAGUUAUATT	UAUAACUGUACACUGCCACTT
siYTHDF2-1	GCCCAAUAAUGCAUAUACUTT	AGUAUAUGCAUUAUUGGGCTT
siYTHDF2-2	GCUCUGGAUAUAGUAGCAATT	UUGCUACUAUAUCCAGAGCTT
siYTHDC2-1	GCGACUCAACAAUGGCAUATT	UAUGCCAUUGUUGAGUCGCTT
siYTHDC2-2	GGAUUUGAUCAUGCAUCUUTT	AAGAUGCAUGAUCAAAUCCTT
siHNRNPC-1	AUUGUUAAGUCAUAAAGAGGUTT	CUCUUUAUGACUUAACAAUAATT
siHNRNPC-2	AAAUAGUAAUUAAAAGAACAATT	GUUCUUUUAAUUACUAUUUAUTT
siMIR4435-2HG-1	AUUUAUGGAUUAAAUUGUGCCTT	CACAAUUUAAUCCAUAAAUCATT
siMIR4435-2HG-2	ACUUAGUUGUAUUUAGAGGAGTT	CCUCUAAAUACAACUAAGUACTT

Table S2. Primer sequences.

Gene (Human)	Forward (5′-3′)	Reverse (5′-3′)
MIR4435-2HG	GGGAAATAAATGACTGGATGG	GAAAGATGCTGGTGACTGC
LINC00271	GCTATTGGTGGGAGGCTTCAG	TGGGCTGGACTTAATGACTTGC
COPDA1	GGTGACGGTGTCGTGGAA	GCAGGTGTAGGTCTGGGTG
MIAT	TTGCAGGAGAGAGAAGTGGG	ACTGGAGGTGAGGCATGAAA
AC005562.1	CACCTCTATAATCCCAGCACT	CTCACTACAGCCTCTGCCTC
FTO	GACCTGTCCACCAGATTTTCA	AGCAGAGCAGCATACAACGTA
YTHDF2	AGCCCACTTCCTACCAGATG	TGAGAACTGTTATTTCCCCATGC
YTHDC2	CAACTCCTAGTAATGAACGGAAGC	TTAAATACTCCTCCTAGTCCAGC
HNRNPC	CCTTACCATCAAACACGATGGC	ACTTCGAAAAGATTGCCTCCACA
β-ACTIN	CTACCTCATGAAGATCCTCACCGA	TTCTCCTTAATGTCACGCACGATT
